# Hereditary Colorectal Cancer Syndromes and Inflammatory Bowel Diseases: Risk Management and Surveillance Strategies

**DOI:** 10.3390/cancers16172967

**Published:** 2024-08-26

**Authors:** Luca Brandaleone, Arianna Dal Buono, Roberto Gabbiadini, Giacomo Marcozzi, Davide Polverini, Michele Carvello, Antonino Spinelli, Cesare Hassan, Alessandro Repici, Cristina Bezzio, Alessandro Armuzzi

**Affiliations:** 1 IBD Center, IRCCS Humanitas Research Hospital, Rozzano, 20089 Milan, Italy; luca.brandaleone@humanitas.it (L.B.); arianna.dalbuono@humanitas.it (A.D.B.); roberto.gabbiadini@humanitas.it (R.G.); giacomo.marcozzi@humanitas.it (G.M.); davide.polverini@humanitas.it (D.P.); cristina.bezzio@hunimed.eu (C.B.); 2 Department of Biomedical Sciences, Humanitas University, Pieve Emanuele, 20072 Milan, Italy; michele.carvello@hunimed.eu (M.C.); antonino.spinelli@hunimed.eu (A.S.); cesare.hassan@hunimed.eu (C.H.); alessandro.repici@hunimed.eu (A.R.); 3 Colon and Rectal Surgery Division, IRCCS Humanitas Research Hospital, Rozzano, 20089 Milan, Italy; 4 Endoscopy Unit, IRCCS Humanitas Research Hospital, Rozzano, 20089 Milan, Italy

**Keywords:** hereditary colorectal cancer syndromes, inflammatory bowel disease, inflammation colon-rectal cancer

## Abstract

**Simple Summary:**

Hereditary colorectal cancer syndromes (HCCS) and inflammatory bowel disease (IBD) carry a high risk of developing colorectal cancer (CRC). The co-occurrence of IBD and HCCS is extremely rare. Individuals with HCCS and IBD are prone to developing CRC at a younger age compared to those without IBD, with patients who have ulcerative colitis at such an especially high risk. The interplay between chronic inflammation and genetic predispositions remains poorly understood.

**Abstract:**

*Background and aims*: Hereditary colorectal cancer syndromes (HCCS), including familial adenomatous polyposis (FAP) and Lynch syndrome (LS), are the two most important high-risk conditions for colorectal cancer (CRC). Inflammatory bowel disease (IBD) increases the risk by two to six times compared with that in the general population. The intersection of these two conditions has rarely been documented in literature. We aimed to summarize the prevalence, pathogenesis, and current evidence-based management of IBD and HCCS and the underlying molecular mechanisms of accelerated carcinogenesis due to combined inflammation and genetic predisposition. *Methods*: PubMed and Scopus were searched until June 2024 to identify relevant studies investigating the epidemiology, pathogenesis, and management of IBD and coexisting hereditary CRC syndromes. *Results*: Co-occurrence of IBD and hereditary CRC syndromes is exceptionally uncommon. Individuals with LS and IBD tend to develop CRC at a younger age than those without IBD, with patients with ulcerative colitis facing particularly elevated risks. The interaction between mismatch deficiency and chronic inflammation requires further investigation.

## 1. Introduction

Colorectal cancer (CRC) is the third most prevalent form of cancer and the second leading cause of cancer-related deaths worldwide [1], with approximately 1,926,118 new cases recorded and an age-standardized incidence rate (ASR) of 12.4% and 9.2 for males and females, respectively [2]. Thus, GLOBOCAN estimates from the International Agency for Research on Cancer (IARC) indicated approximately 903,859 deaths attributed to colorectal cancer and an age-standardized mortality rate (ASRm) of 5.5% and 4% for males and females, respectively [2,3,4]. 

Hereditary colorectal cancer syndromes (HCCS) and inflammatory bowel diseases (IBD), including Crohn’s disease and ulcerative colitis, are the two most frequent non-modifiable risk factors for colorectal cancer [5,6]. Hereditary colorectal cancer syndromes, such as Lynch syndrome (LS) and familial adenomatous polyposis (FAP), are caused by mutations and defects in specific cancer genes, contributing up to 10% of all CRC [7]. Early onset of adenomatous polyps is, to some extent, the hallmark of both FAP and LS. In contrast to sporadic colorectal cancer, the well-known adenoma–carcinoma sequence has been shown to be accelerated in individuals affected by HCCS [7,8,9]. Consequently, these inherited syndromes are associated with a significant lifetime risk of developing CRC, which is estimated to be between 70 and 100 percent for familial adenomatous polyposis [10]. Conversely, patients with LS display variable colorectal cancer lifetime risks based on the involved and defective mismatch repair (dMMR) gene. The observed lifetime risks of colorectal cancer for MLH1/MSH2 gene mutation carriers range from 22 to 74% (average 30%), as well as for mutated MLH6 in male patients [8], while being lower than 20% for PMS2 [11].

Patients with IBD have a two- to six-fold increased risk of developing colorectal cancer compared to the general population [12,13], and this risk has been established to grow proportionally with disease duration [14]. Therefore, the European Crohn’s and Colitis Organization (ECCO) recommends surveillance colonoscopy starting from eight years after the onset of symptoms in all patients with colonic IBD, and a tailored endoscopic surveillance strategy is recommended [15] on the basis of a colorectal cancer risk stratification [15]. 

Individuals with IBD may be at risk of developing primary sclerosing cholangitis (PSC), with a pooled prevalence of 2.1%, according to a recent meta-analysis [16]. PSC is a significant risk factor for both hepatobiliary and colorectal cancers [17]; hence, as recommended by the European Crohn’s and Colitis Organization and European Association for the Study of the Liver (EASL), surveillance colonoscopy in patients with IBD-PSC should be performed annually [18,19]. 

LS or FAP in conjunction with IBD is an extremely rare phenomenon and might increase the risk of CRC and early-onset CRC. This overlap is scarcely documented in the scientific literature, with only a few case reports and series. This review aims to examine the epidemiology, risk of CRC, and surveillance strategies for concomitant hereditary colorectal syndromes and IBD, with a focus on the potential mechanisms of accelerated carcinogenesis and prognosis.

## 2. Materials and Methods

PubMed/MEDLINE and Scopus databases were searched until June 2024 to identify relevant studies investigating the coexistence of IBD and hereditary colorectal cancer syndromes. The following text words and corresponding Medical Subject Heading/Entree terms were used: “inflammatory bowel disease” OR “IBD” OR “ulcerative colitis” OR “Crohn’s disease” AND “Lynch syndrome” OR “familial adenomatous polyposis” OR “hereditary colorectal cancer”. Additional publications were identified by a manual search of abstracts from the annual meetings of Digestive Disease Week, American College of Gastroenterology, European Crohn’s and Colitis Organization, and United European Gastroenterology Week. No restrictions on publication date were imposed. Both animal and human studies were included. 

## 3. Overlapping Lynch Syndrome and IBD

During the last twenty years, both cases of IBD with overlapping FAP or LS have been described. Most cases were identified after the diagnosis of malignancy and/or as a consequence of a highly suspicious family history, and some of them received an intraoperative diagnosis.

Table 1 elucidates all available studies investigating the clinical features and outcomes of patients with co-existing IBD and HCCS.

### 3.1. IBD and Overlapped Lynch Syndrome

Lynch syndrome is estimated to be responsible for 3–6% of hereditary colorectal cancer cases [7,25]. The Amsterdam Criteria II [26] and Revised Bethesda Criteria [27] represent two pivotal clinical tools that guide physicians in recognizing LS nuclear families. Nevertheless, definitive diagnosis of LS requires molecular identification of pathological or likely pathogenic variants that result in defective mismatch repair genes (dMMR) [28]. Win et al. [29] estimated that 1 in 279 (0.359%) individuals may be carriers of pathological or likely pathological variants of any MMR genes based on population-based cancer registers. In subgroup gene analysis, MLH1 and MSH6 exhibited the highest population prevalence (1 in 714 (0.140%) vs. 1 in 758 (0.132%)) [29]. 

Inflammatory bowel disease (IBD), such as Crohn’s disease and ulcerative colitis, are chronic, debilitating inflammatory conditions that result in increased healthcare costs [30,31]. A recent systematic analysis revealed an elevated estimate of prevalence and total number of deaths associated with IBD over time, from 1990 to 2019 [32]. Ng et al. [33] conducted a systematic review of the incidence and prevalence of inflammatory bowel disease. Researchers observed the highest prevalence values in northern European countries, such as Norway and Germany, which reported an incidence of 505 cases per 100,000 for ulcerative colitis and 322 per 100,000 for Crohn’s disease [33,34]. 

In 1999, Matsuda et al. reported two cases of CRC in patients with a history of both IBD and familial CRC [35]. The first patient was a 41-year-old woman who had undergone a total colectomy with ileo-rectal anastomosis (IRA), who under the following regular endoscopic surveillance developed high-grade dysplasia on the biopsies of the rectal stump, making it necessary to carry out surgery with ileo-anal anastomosis (IAA) [35]. Histology revealed submucosal invasive carcinoma, and immunohistochemistry (IHC) revealed p53 overexpression (Table 2). The patient’s family history fulfilled the Amsterdam Criteria [36] for hereditary non-polyposis colorectal cancer (HNPCC). The second patient was a 47-year-old man with ulcerative colitis (UC) who had undergone subtotal colectomy with ileorectal anastomosis (IRA) the following year. Endoscopic surveillance after one year revealed a dysplastic lesion, leading to removal of the rectal stump with IAA. Similarly, patients’ family history presented > 2 cases of CRC on the maternal side as well as other Lynch-associated tumors (i.e., stomach, uterus, and brain) [35]. 

In 2014, Minani et al. reported a case of a 28-year-old woman with UC who showed severe endoscopic disease activity under endoscopic surveillance [Mayo endoscopic score (MES) 3 in the rectum], as well as numerous polyps throughout the whole colon, including a 0-Isp polyp with a central depression that resembled an inflammatory polyp. Histological examination revealed signet ring cell carcinoma with cryptal distortion and lymphoid aggregation, indicating chronic inflammation. The patient was diagnosed with colitis-associated colorectal cancer related to UC and underwent a total proctocolectomy. Four years later, her sister and cousin were diagnosed with LS and were found to have an MSH2 mutation after germline testing. Later, the patient underwent surgery for endometrial and ovarian cancer [37].

In 2022, Ayeni et al. [38] presented a case of a 32-year-old male patient with ulcerative colitis (UC) and Lynch syndrome, a hereditary condition associated with a mutation in the MSH6 gene. The patient was treated with mesalamine and steroids until an unresectable low-grade dysplastic tubulovillous adenoma was detected during endoscopic surveillance. Subsequently, the patient underwent hemicolectomy. However, during endoscopic surveillance after surgery, multiple rectal and sigmoid tubular adenomas with low-grade dysplasia were identified, prompting recommendations for further surgical intervention (Table 2). Notably, histology revealed the presence of adenocarcinoma arising from tubule-villous adenoma, with a minimum of twenty-one foci exhibiting carcinomatous transformation across the entire large bowel [38].

Currently, a retrospective cohort study is ongoing in the U.S. aiming to compare the prevalence of CRC in individuals with LS and concomitant IBD vs. LS alone and to evaluate factors associated with CRC [24]. The preliminary data of this study (LS and IBD, *n* = 569) showed a significantly higher prevalence of CRC, small bowel cancer, and colorectal polyps in this subset (*p* < 0.0001) than in LS alone (*n* = 24.584). In the IBD + LS subgroup, the estimated adjusted odds ratios for small bowel cancer, colonic polyps, and colorectal cancer were 2.66 (95% CI, 1.58–4.47), 1.46 (95% CI, 1.21–1.76), and 1.55 (95% CI, 1.10–2.18), respectively. Therefore, IBD, specific genotypes (MLH1, MSH2, EPCAM), and advancing age were found to be independent risk factors for neoplasia, indicating that patients with LS who have these risk factors might benefit from more intensive surveillance [24]. Table 2 summarizes the main clinical, endoscopic, histologic, and molecular features of CRC in HCCS, in IBD and in HCCS + IBD.

### 3.2. IBD and Overlapped FAP

In 2006, Fukushima et al. [39] reported a case of a young man affected by FAP with no familial history of familial adenomatous polyposis or colorectal cancer, who initially intended to undergo total proctocolectomy and ileal- J pouch-anal anastomosis. Under a laparotomic view, wall thickening and stricture of the terminal ileum, 30 cm in length, were observed; thus, an ileostomy was constructed. The histology was suggestive of Crohn’s disease [39]. 

Two patients with a history of HCCS associated with pouchitis and fistulizing perianal disease, who ultimately had CD, were reported by Gentile et al. [40]. Both cases were diagnosed during the postoperative follow-up with CD after several histological re-evaluations upon persistent evidence of ileal thickening on CT scans [40].

In 2013, a 59-year-old man with a family history of familial adenomatous polyposis and colorectal cancer was referred to a gastroenterologist due to increased stool frequency, urgency, and occasional rectal bleeding. Colonoscopy revealed several sessile polyps in the right colon, and acute inflammation resembling ulcerative colitis in the left colon. Histology of the right colon specimen showed adenoma, whereas the left colon specimens were consistent with ulcerative colitis. DNA sequence analysis of APC from both the right and left colon revealed a 426–427 delAT mutation, confirming the genetic diagnosis of FAP [41].

More recently, a case series was conducted as part of the ECCO Collaborative Network of Exceptionally Rare Case Reports (CONFER) project, which included 26 individuals with IBD (10 with UC, 15 with CD, and one with U-IBD) and HCCS [23]. Among the included patients, 42.3% were diagnosed with HCCS prior to IBD, the remaining 42.3% were diagnosed with IBD prior to HCCS, and four patients (15.4%) were diagnosed with both conditions simultaneously. In this study, sixteen patients exhibited LS, seven had familial adenomatous polyposis (FAP), two had MYH-associated polyposis (MAP), and one had attenuated FAP (AFAP). The most prevalent genetic mutations were APC (*n* = 7) and MLH1 (*n* = 7). Ten (38.5%) participants developed colorectal cancer (CRC), four developed CRC after being diagnosed with IBD, four developed CRC before being diagnosed with IBD, and two developed CRC at the same time as their IBD diagnosis [23].

## 4. Mechanisms of IBD-Associated CRC

The development of CRC is contingent upon two principal events occurring in sequence: firstly, a driver event, which involves the accumulation of genetic or epigenetic mutations that result in replicative and survival advantages. Secondly, tumor promotion must occur, which entails the clonal expansion of altered cells leading to the formation of a frank tumor [19].

### 4.1. Colitis-Associated Colorectal Cancer (CAC) Occurrence

Similarly, colitis-associated colorectal cancer (CAC) necessitates an initiating event such as chronic inflammation resulting from inflammatory bowel disease [12,42,43]. Therefore, at least two mechanisms are likely to influence carcinogenesis and tumor progression. The first mechanism is related to chronic bowel inflammation, which may be secondary to environmental factors (e.g., infections, smoking) or dietary habits, as previously described by Newmark et al. [44]. Inflammation can initiate tumorigenesis via DNA damage and accumulation of mutations in oncogenes, even in the absence of exogenous factors [45]. Notably, the increased levels of reactive species (that is, ROS, RNS) are responsible for DNA damage in intestinal epithelial cells (IECs) [46]. Consequently, impaired bowel barrier function contributes to carcinogenesis by interacting with IECs, intestinal stem cell compartments, and intestinal microbiota, leading to persistent activity of the mucosal immune cell system and fueling chronic bowel inflammation [47,48]. In contrast, chronic inflammation leads to tumor promotion and dedifferentiation of non-stem cells into stem-like cells [49]. Notably, interleukin-6 (IL-6) appears to play a pivotal role in chronic inflammation by suppressing neutrophil infiltration and stimulating either the adaptive immune system or carcinogenesis [50]. Interleukin-6 activates several cytokines, such as NF-κB and STAT3, which induce the expression of oncogenes, cell death, and immune response [51]. Thus, IL-6 is crucial for the development of colorectal cancer [52,53]

### 4.2. Chromosomal Instability: The Adenoma–Carcinoma Sequence

The second mechanism is related to tumor microenvironment (TME) and cellular interactions within the TME, eliciting inflammation. Dysregulation of the Wnt/β-catenin signaling pathway in intestinal epithelial cells results in a loss of gut barrier function, leading to upregulation of IL-17 and IL-23 by myeloid immune cells [54]. Epithelial tumor cells and resident immune cells secrete various cytokines such as IL-1, which interact with IECs and T-cells, thereby accelerating carcinogenesis [55]. Finally, the loss of p53 during tumor progression is associated with increased intestinal permeability, causing the formation of an NF-κB-dependent inflammatory microenvironment [56]. TP53 mutations appear to occur earlier during carcinogenesis in colitis-associated colorectal cancer [43].

In contrast to sporadic colorectal cancer, colitis-associated colorectal cancer is thought to arise from different dysplastic foci, where the inflamed colonic mucosa undergoes cancer-related cancer-associated molecular alterations, resulting in epithelial dysplasia [57]. At the molecular level, colorectal cancer development and pathogenesis can be attributed to at least three primary pathways. The conventional chromosomal instability (CIN) pathway, which accounts for approximately 70–80% of cases, is initiated by APC mutations, followed by mutations in K-RAS, PIK3CA, and SMAD4 [3,58,59].

Hence, loss of heterozygosity on chromosome 18 (loH 18q) results in TP53 mutations and defines the adenoma–carcinoma sequence, as previously described by Vogelstein [60]. Notably, it is observed that mutations in APC and K-RAS genes are seen less frequently in colitis-associated colorectal cancer [61]. It is hypothesized that these mutations arise later in the dysplasia–carcinoma sequence.

Finally, approximately 20–30% of CRC cases develop through the serrated pathway, which includes the activated MAPK pathway and CIMP mutations. 

### 4.3. Microsatellite Instability (MSI)

**Figure 1:**
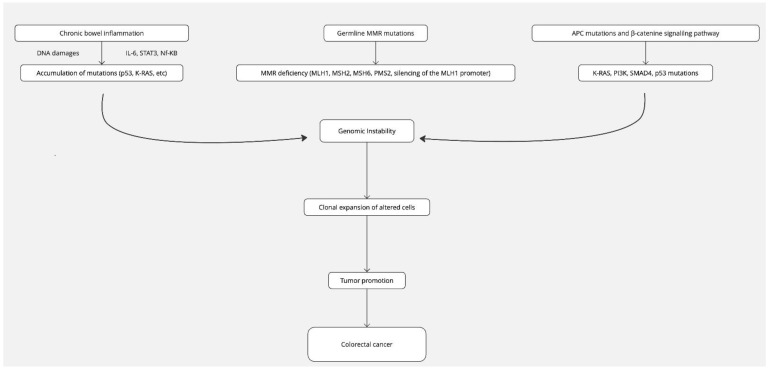
Proposed molecular mechanism of colorectal carcinogenesis in coexisting IBD-HCCS. MMR: mismatch repair.

Microsatellites, also referred to as short tandem repeats (STRs), are composed of repeated sequences of 1–6 nucleotides [62]. Defective MMR genes (MLH1, MSH2, MSH6, PMS2, and silencing of the MLH1 promoter) are associated with microsatellite instability (MSI) (Figure 1) [62]. 

MSI is responsible for approximately 15% of colorectal cancer (CRC) cases, including 3% of inherited CRC cases and 12% of noninhibited CRC cases [62,63].

Sporadic CRC with MSI occurs via sporadic methylation-induced silencing of the MLH1 promoter as a result of epigenetic mutations on clustered cytosine-guanosine residues called CpG islands [64]. This epigenetic process is associated with advanced age and chronic inflammations [65]. When the MLH1 promoter is methylated, MMR activity fails and MSI ensues [66,67]. Other characteristic features of sporadic CRC with MSI include frequent mutations (usually V600E) in BRAF and absence of MLH1 and PMS2 proteins [68]. Notably, it is possible to recognize two different CRC subtypes on the basis of BRAF V600E: BRAF mutant 1 (BM1), associated with activation of K-RAS/AKT pathway, dysregulation of mTOR/4EBP, and activation of epithelial to mesenchymal transition (EMT); BRAF mutant 2 (BM2) is distinguished by dysregulation of the cell cycle checkpoints, so it exhibits low levels of cyclin D1 (CD1) but increased CD1-kynase (CDK1) levels [69]. In comparison to microsatellite stability (MSS), microsatellite instability (MSI) tumors are more frequently located in the proximal colon, display a poor differentiation, and exhibit a mucinous or signet ring histological type, as previously described [70,71].

The Colorectal Cancer Subtyping Consortium (CRCSC) recently defined a prognostic classification based on molecular features [72]. Consequently, CRC is subdivided into four distinct subtypes: the classification system identifies four distinct subtypes: CMS1 (MSI-Immune), CMS2 (Canonical), CMS3 (Metabolic), and CMS4 (Mesenchymal). CMS-1 represents up to 15% of all CRC cases and is distinguished by microsatellite instability, high CIMP positivity, hypermethylation, and somatic copy number alterations (SCNA)-low, as well as BRAF and TGFBR2 mutations [72,73,74]. The most prevalent molecular CRC subtype was CMS-2, accounting for 37% of cases. Its defining characteristics include microsatellite stability (MSS), chromosomal instability (CIN) at a high level, CIMP negativity, SCNA at a high level, and mutations in APC and TP53 genes [72,74]. CMS-3 is distinguished by metabolic deregulation, mixed microsatellite status (CIN-intermediate, CIMP-low, SCNA-intermediate), K-RAS, and APC mutations. In conclusion, CMS-4 is associated with stromal infiltration, TGF-β activation, EMT activation, angiogenesis, microsatellite stability, CIN-high, CIMP-negative, SCNA-high [72,73]. 

LS-related tumors result from germline mutations in DNA mismatch repair (MMR) genes (for example, MLH1, MSH2, PMS2, and MSH6), which give rise to a hypermutagenic MSI phenotype [75]. To ensure accurate DNA replication, the MMR gene system forms heterodimers, consisting of MutSα and MutLα, which work together to detect and repair these errors. MutSα, aided by MSH6, binds to DNA at mismatch sites, whereas MutLα, aided by PMS2, marks repair sites [76]. Finally, loss of function (LoF) in the MMR system can have detrimental effects on genomic integrity and contribute to cancer development [67,71,75,77]. 

## 5. CRC Risk of Concomitant IBD and HCCS

IBD and HCCS are the most significant acquired and inherited risk factors for the development of CRC. However, the risk of developing CRC in patients affected by both conditions remains unclear, with some emerging recent data. While the exact risk of CRC in individuals with both conditions is unclear, there is evidence suggesting an increased risk compared to that in the general population, as reported in several case studies. Derikx et al. [21] compared the risk of CRC between two distinct groups: LS-IBD (*n* = 15) and LS (*n* = 1031). Although the prevalence of colorectal cancer was equal in both groups, patients diagnosed with both LS and IBD (LS-IBD) were significantly younger than those with Lynch syndrome (median age 38.0 vs 52.0 years; *p* = 0.001). All the patients in the LS-IBD group who developed CRC were affected by ulcerative colitis, resulting in a higher cumulative CRC incidence (*p* < 0.001). Despite the identification of four LS-related adenocarcinomas in the IBD-LS group, the proportion of participants with multiple CRCs was not significantly different between the cases and controls (*p* = 0.205). 

Similarly, in 2016, McNamara et al. [20] identified and collected clinical data from a Canadian registry for all cases of co-existing inflammatory bowel disease and Lynch syndrome. Twelve confirmed cases were identified, and four cases of CRC were observed in this group. In particular, LS was diagnosed concurrently with colorectal cancer in three out of four patients, while Lynch syndrome was confirmed subsequent to an early onset CRC diagnosis from a family member [20]. Consequently, the patients underwent colectomy. Four additional colectomies were performed in the IBD-LS group. Of these, one was performed for severe colitis, whereas the other three were performed for dysplasia. All colectomies were performed in patients with long-standing ulcerative colitis. 

A recent study by Faisal et al. [22] evaluated the impact of inflammatory bowel disease on a cohort of individuals with Lynch syndrome and compared the proportion of patients with cancer in the LS group with and without IBD. Twenty-one patients with LS and IBD were compared with the control LS group (*n* = 43). The researchers observed that LS-specific cancers were diagnosed in 52.4% of the IBD-LS group compared to 44.2% of the control LS group (*p* = 0.54). Among the seven individuals with IBD (five with Crohn’s disease and two with ulcerative colitis), four (57.1%) developed colorectal cancer (CRC) over a median 10-year period from IBD diagnosis, compared to 53.5% of the control group (*n* = 43) when followed for 10 years (*p* = 0.86) [22].

In conclusion, HCCS and IBD represent the two most significant risk factors for the development of colorectal cancer. The co-existence of disease is a rare phenomenon, as evidenced in the medical literature. The precise risk of developing CRC remains unclear. Endoscopic surveillance is a mandatory procedure for all individuals with a genetic predisposition to colorectal cancer and those at high risk of developing the disease, in accordance with the most recent guidelines (Table 3).

## 6. Discussion

This review outlines the available scientific work on patients with the rare condition of concomitant IBD and HCCS. Numerically, the studies that have investigated this condition have mainly included patients with LS, while records with hereditary polyposis (e.g., FAP, aFAP, and MAP) are rarer, also and above all because of the infrequency of the latter conditions. Similarly to as in the absence of IBD, in the case of LS, the most prevalent mutation in the overlap population concerns MLH1, just as in the case of polyposis the most frequently defective gene is APC also in IBD-polyposis populations [16,20,21,24]. 

According to the data presented, the diagnosis of IBD mostly precedes the diagnosis of HCCS, implying that HCCS should be considered in patients with IBD who develop CRC, particularly if they are young at the time of CRC diagnosis and/or in presence an evocative family history. The cumulative CRC risk in the IBD-HCCS population has been estimated as higher (OR~1.55–2.0) as compared to both conditions alone [21,24]. The concomitance of IBD with LS with a specific PMS2 mutation confers to this syndrome, which is normally much more indolent within the Lynch spectrum, with oncological risks more similar to those observed for more aggressive and penetrating genotypes (e.g., MLH1, MSH2). 

Among extra-intestinal cancers’ risk, preliminary data suggest a significantly in-creased risk of small bowel cancers especially in IBD with LS and specifically associated with defects in MLH1 and MSH6. 

Regarding the analysis per sub-groups of IBD, UC, and CD, data are extremely scanty; however, according to Derikx et al., CRC specifically developed more frequently in LS patients with UC as compared to CD [21]

The biological basis of this amplified carcinogenesis and compounded CRC risk may be due to the interaction between MMR deficiencies and inflammation: this latter can cause DNA damage and impair even more or suppress MMR responses (Figure 1). This hypothesis has been described in MSH2 biallelic defective mice, but the demonstration failed in the case of heterozygosity where no increased tumor susceptibility over wild-type genotype was observed [80]

HT Lynch himself suggested that that many members of the LS family that he described might possibly inherit a major gene that predisposes them to the disease, causing them to be carriers of a subclinical form of IBD with minimal morphological markers [81]. The authors also postulated that this condition might become apparent only in some family members when additional factors come into play [81]. Together with HT Lynch, other authors have hypothesized that the gene/s responsible for developing IBD might provide protection against LS, potentially resulting in a selective genetic advantage [81,82]. Indeed, a significant association between IBD and specific haplotypes of MLH1 has been reported, emphasizing that a deficient MMR system can lead not only to carcinogenesis, but importantly also to the production of aberrant protein products able to initiate an autoimmune response [83].

**Figure 2:**
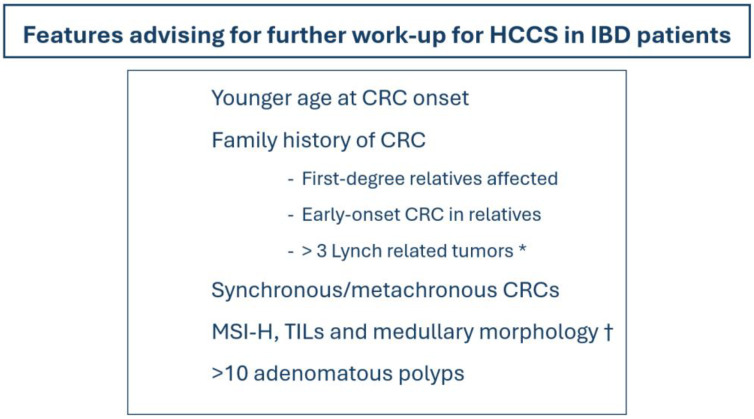
Clinical clues evoking further work-up for HCCS are needed in IBD patients. * stomach, uterus, ovary, small bowel, urinary tract cancers; † medullary CRC sub-type. HCCS: hereditary colorectal cancer syndromes; IBD: inflammatory bowel diseases; CRC: colorectal cancer; MSI-H: microsatellite instability high; TILs: tumor-infiltrating lymphocytes.

With respect to histopathology, CRC occurring in IBD patients are distinguished by tumor histologic heterogeneity, despite a mostly well-differentiated, Crohn-like reaction, the presence of mucin, and signet ring cell differentiation [84]. These features are shared by both sporadic and LS-associated MSI-high CRCs, hinting at possible similar and shared genetic alterations as well as molecular pathways in IBD and LS patients [84]. Usually, the occurrence of an MSI-H profile in IBD-related CRCs is typically attributed to the hypermethylation of the MLH1 gene promoter, which leads to the loss of MLH1 protein expression [85]. These morphological observations advise against an age-based and morphology-based screening for LS in IBD patients, which may be less effective in this population than compared to non-IBD individuals. In Figure 2, we provide clear clinical clues for referring IBD patients for the work-up of LS and HCCS.

## 7. Conclusions

The optimal medical approach in patients with co-existent IBD and HCCS is still to be demonstrated and standardized and warrants data from real-world cohorts. The occurrence of CRC in a patient with IBD and concomitant HCCS enforces colectomy due to the high risk of recurrence and of developing multiple metachronous CRCs. Future research will determine whether patients with IBD-HCCS can benefit from more stringent and customized endoscopic surveillance, and whether a stratification for UC/CD and for disease duration can be pursued to tailor intervals and techniques. Finally, the use of immunomodulators in patients with HCCS and IBD needs to be addressed by future studies in order to understand to what extent the drug-induced impairment of immunosurveillance is involved in digestive cancers in these sub-group of patients.

## Figures and Tables

**Table 1:** Studies investigating clinical outcomes of patients with co-existing IBD and Lynch Syndrome.

Studies Investigating Clinical Outcomes of Patients with Co-Existing IBD and Lynch Syndrome (LS)								
Authors	Year	Country	IBD + LS (*n*)	LS (*n*)	CRCs (*n*)	IBD-LS Related CRC (%)	LS-Related CRC (%)	*p*-Value
McNamara et al. [20]	2016	United States	12		4	33.3% (4/12)		
Derikx et al. [21]	2017	Netherlands	15	1031	315	26.7% (4/15)	30.2% (15/1031)	0.205
Faisal et al. [22]	2022	United States	7	50		57.1% (4/7)	53.5% (23/43)	0.86
Barberio et al. [23]	2022	Italy, France, Belgium, Israel, Germany, Malta	16			50% (8/16)		
Patel S et al. [24]	2024	United States	568	24,016	1277	7.7% (44/567)	4.9% (1183/25,306)	<0.001

**Table 2:** Main clinical, endoscopic, histologic, and molecular features of CRC in HCCS, in IBD, and in HCCS + IBD.

	CRC Location	Endoscopic Features	Histologic Features	Molecular Pathway
HCCS	In LS, CRC are typycally located in the right colon	Poypoid and non-polypoid lesions; multiple/multifocal	CRC can display a poor differentiation, and exhibit a mucinous or signet ring histological type	In LS mostly via microsatellite instability (MSI)
LS, FAP, aFAP, MAP	In polyposis, syndromes can be located in any segment (left > right)			In polyposis, syndromes displays chromosomal instability (via inactivation of APC, and overexpression of β-catenin)
IBD (UC, CD)	Can be located in any segment, usually linked to disease extension (left > right)	Polypoid, non-polypoid, or invisible; inflammatory changes can be present; stricturing, ulcerated, irregular; multifocal	Mucinous, usually moderately differentiated to signet ring cell with/without inflammatory component	Inflammation-dysplasia-carcinoma sequence; aneuploidy, TP53, APC, and KRAS mutations
HCCS + IBD	Can be located in any segment, can present multiple neoplasia (synchronous/metachronous)	Mixed and heterogeneous aspects; polypoid, non-polypoid, or invisible; multifocal	From mucinous (moderately differentiated) to partly signet ring cell carcinoma (poorly differentiated), or micropapillary, partly mucinous	Unknown; possible DNA damage linked to inflammation, with worsened impairment/suppression of MMR

HCCS: hereditary colorectal cancer syndromes; CRC: colorectal cancer; LS: Lynch syndrome, FAP: familial adenomatous polyposis (classical phenotype); aFAP: attenuated familial adenomatous polyposis; MAP: MUTYH-related adenomatous polyposis.

**Table 3:** Endoscopic surveillance for genetic and high-risk CRC populations.

Surveillance Recommendations for Genetic and High-Risk CRC Individuals				
	Age to Begin Surveillance (Years)	Surveillance Interval (Years)	Surveillance Procedures	Reference
**Inflammatory Bowel Diseases**				
	6–8 after symptoms onset	1–3–5 according to risk stratification	Colonoscopy	[15,78]
**Hereditary Colorectal Cancer Syndrome**				
**Lynch Syndrome**				
MLH1	20–25	1–2 ^a^	Colonoscopy	[7]
	20–25 ^b^	1–2	Colonoscopy	[79]
MSH2	20–25	1–2	Colonoscopy	[7]
	20–25 ^b^	1–2	Colonoscopy	[79]
MSH6	25–30	1–2	Colonoscopy	[7]
	30–35 ^c^	1–3	Colonoscopy	[79]
PSM2	25–30	1–2	Colonoscopy	[7]
	30–35 ^c^	1–3	Colonoscopy	[79]
**Familial Adenomatous Polyposis**				
APC	10–15	1–2	Flexible RSS or Colonoscopy ^d^	[7]
	10–15	1	Colonoscopy	[79]

a: Consider annual colonoscopy in confirmed mutation carriers; b: 2–5 years prior to the earliest CRC if it is diagnosed before age 25 years; c: –5 years prior to the earliest CRC if it is diagnosed before age 30 years; d: annual colonoscopy if surgery is delayed for >1 year after onset of colon polyps.

## Abbreviations

HCCS: hereditary colorectal cancer syndromes; HNPCC: hereditary non-polyposis colorectal cancer; IBD: inflammatory bowel disease; UC: ulcerative colitis; CD: Crohn’s disease; MES: Mayo endoscopic score; CRC: colorectal cancer; CAC: colitis-associated colorectal cancer; MMR: mismatch repair; FAP: familial adenomatous polyposis; AFAP: attenuated familial adenomatous polyposis; MAP: MUTYH-associated polyposis; MSI: microsatellite instability; CMS: consensus molecular subtype; IECs: intestinal epithelial cells; TME: tumour microenvironment; IAA: ileo-anal anastomosis; IRA: ileo-rectal anastomosis; ROS: reactive oxygen species; RNS: reactive nitrogen species.
